# Pre- and post-dilatation in transcatheter aortic valve implantation with self-expanding valves: a systematic review and meta-analysis

**DOI:** 10.3389/fcvm.2026.1883882

**Published:** 2026-08-26

**Authors:** Bing Wei Thaddeus Soh, Ailís Pollock, Dominic Butler, Angela McInerney, John William McEvoy, Darren Mylotte

**Affiliations:** 1 Department of Cardiology, University Hospital Galway, Galway, Ireland; 2 School of Medicine, University of Galway, Galway, Ireland; 3 Department of Cardiology, Tallaght University Hospital, Dublin, Ireland

**Keywords:** balloon valvuloplasty, post-dilatation, pre-dilatation, self-expanding valve, TAVI

## Abstract

**Introduction:**

Pre-dilatation balloon aortic valvuloplasty (PRE-BAV) and post-dilatation (POST-DIL) are commonly used during transcatheter aortic valve implantation with self-expanding valves (SEV-TAVI) to facilitate device delivery and optimize valve expansion, but both may increase procedural complications.

**Methods:**

We conducted a systematic review and meta-analysis to evaluate outcomes associated with PRE-BAV and POST-DIL compared with direct implantation (DIRECT) and valves left as deployed (AS-DEPLOYED), respectively. Eleven studies (nine observational, two randomised; 24,143 patients) were included for PRE-BAV and five observational studies (7,526 patients) for POST-DIL. Key outcomes included mortality, stroke, permanent pacemaker implantation, and moderate or greater paravalvular leak (PVL2+). Random-effects meta-analyses with Hartung–Knapp–Sidik–Jonkman adjustment were used to estimate pooled odds ratios (ORs) with 95% confidence intervals (CIs).

**Results:**

Compared with DIRECT, PRE-BAV was not associated with differences in mortality, stroke, pacemaker implantation, or PVL2+, but randomised data showed a reduced need for POST-DIL (OR 0.43; 95% CI 0.24–0.74). Compared with AS-DEPLOYED, observational series suggested that POST-DIL was linked to higher 30-day mortality (OR 1.39; 95% CI 1.18–1.63), increased PVL2+ (OR 3.76; 95% CI 2.13–6.63), and more frequent surgical conversion (OR 1.22; 95% CI 1.06–1.41), with no differences in stroke or pacemaker implantation.

**Discussion:**

PRE-BAV was not associated with increased adverse outcomes and may reduce the need for post-dilatation, whereas the adverse outcomes observed with POST-DIL in observational studies are most likely explained by confounding by indication rather than a causal effect. Further adequately powered randomised trials are needed to define the optimal balloon dilatation strategy in SEV-TAVI.

**Systematic Review Registration:**

https://www.crd.york.ac.uk/PROSPERO/view/CRD42024610604.

## Introduction

Pre-dilatation balloon aortic valvuloplasty (PRE-BAV) prior to transcatheter heart valve (THV) implantation was historically a routine step in transcatheter aortic valve implantation (TAVI) (1, 2), but its use has significantly declined in contemporary practice (3–7). This change in practice has been driven by concerns regarding PRE-BAV related complications, including haemodynamic instability during balloon inflation (8), aortic annular injury (9), and increased risks of permanent pacemaker (PPM) implantation and stroke (10, 11), as well as efforts to streamline procedures and reduce procedural time and costs through direct THV deployment without pre-dilatation (DIRECT) (3). While omission of PRE-BAV may be less consequential for balloon-expandable valves (BEV) deployed under controlled balloon expansion, it may have more important implications in self-expanding valve (SEV) TAVI, where valve expansion relies solely on intrinsic radial force.

Balloon post-dilatation (POST-DIL) is performed after THV deployment to optimize frame expansion, reduce paravalvular leak (PVL), and enhance haemodynamics (2). However, contemporary data show its use in only 14%–23% of TAVI procedures (5, 7), with the majority of valves being deployed without undergoing further POST-DIL (AS-DEPLOYED). This selective use likely reflects concerns about POST-DIL complications such as aortic annular rupture (12), stroke (13), mortality (14), THV embolization (15, 16), and potential damage to bioprosthetic valve leaflets (17). However, much of the evidence linking POST-DIL to adverse outcomes comes from small retrospective observational studies, which are often underpowered, and include both BEVs and SEVs, potentially obscuring the true effect of POST-DIL in SEV alone, and are further limited by confounding by indication, as POST-DIL is typically performed in response to suboptimal valve deployment (10, 12–14).

Recent studies have highlighted concerns regarding SEV malexpansion, which has been associated with worse haemodynamics and clinical outcomes, including higher post-procedural gradients, structural valve deterioration, and greater incidence of PVL, stroke, rehospitalization, and mortality (18–21). Malexpansion has also been associated with hypoattenuated leaflet thickening (HALT), a phenomenon associated with increased thrombotic risk and potential long-term valve dysfunction (22, 23). In this context, both PRE-BAV and POST-DIL are key adjunctive procedures to optimize THV deployment by preparing the annulus and correcting residual frame malapposition.

Despite this, randomised controlled trial (RCT) data are limited, and procedural decision-making during SEV-TAVI remains highly variable. The role of balloon dilatation in preventing or treating valve malexpansion therefore remains uncertain. This uncertainty is compounded by procedural variation and, particularly for POST-DIL, by its use in response to suboptimal valve deployment. We therefore performed a systematic review and meta-analysis to evaluate the impact of PRE-BAV and POST-DIL on clinical and haemodynamic outcomes in SEV-TAVI.

## Methods

### Study design

This systematic review and meta-analysis adhered to the Preferred Reporting Items for Systematic Reviews and Meta-Analyses (PRISMA) guidelines (Supplementary Table S1) (24), and was registered in the International Prospective Register of Systematic Reviews (PROSPERO) before commencement (CRD42024610604) (25). Ethical approval was not required, as the study analysed only previously published data without direct patient involvement.

### Search strategy

A systematic search was conducted across the Medline, Embase, Scopus, and Cochrane databases on November 18, 2024, using the following keywords and MeSH terms (Full search strategy available on Supplementary Table S2): (1) aortic valve stenosis OR aortic stenosis; (2) transcatheter aortic valve replacement OR TAVR OR transcatheter aortic valve implantation OR TAVI; (3) CoreValve OR Evolut OR Evolut R OR Evolut PRO OR Evolut PRO + OR ACURATE neo OR ACURATE neo2 OR Portico OR Allegra OR self-expanding valve OR self-expanding supraannular valve; (4) pre dilatation OR pre-dilatation OR pre dilation OR pre-dilation OR post dilatation OR post-dilatation OR post dilation OR post-dilation OR malexpansion OR incomplete expansion OR underexpansion OR suboptimal valve expansion OR frame deformation OR distorted valve frame OR non-uniform deployment OR irregular radial expansion; (5) Combination: (1 AND 2 AND 3 AND 4). Additionally, a manual hand search was performed to identify relevant studies involving the Navitor valve, along with screening of reference lists from included articles.

### Study selection

After removing duplicates, two authors (BWTS and AP) independently screened titles and abstracts for relevance and eligibility. The dual independent review process assessed all qualifying full-text articles against predefined inclusion and exclusion criteria. Studies were included if they (1) involved adult patients undergoing TAVI with SEVs for aortic stenosis; (2) assessed PRE-BAV or POST-DIL techniques during SEV-TAVI through comparative analysis with a no balloon dilatation control group (DIRECT or AS-DEPLOYED); (3) reported clinical outcomes (e.g., mortality, stroke, complications, PPM implantation), echocardiographic outcomes (e.g., PVL, mean gradient, effective orifice area), or measures of valve malexpansion; (4) were RCTs, cohort studies, case-control studies, or other observational studies published as full-text articles in peer-reviewed journals; and (5) were published in English.

Studies were excluded if they (1) focused solely on TAVI using non-SEVs without relevant SEV data; (2) did not investigate PRE-BAV or POST-DIL techniques; (3) did not report on clinical, echocardiographic, or malexpansion-related outcomes; (4) were abstracts, conference posters, letters to the editor, commentaries, reviews, or non-peer-reviewed articles; or (5) were not published in English. All discrepancies between the two authors were resolved through consultation with a third author (DM).

### Data extraction

Two authors (BWTS and AP) independently extracted data from the main text and supplementary materials of the included studies. Discrepancies were resolved through consultation with a third author (DM). The extracted data included study design and characteristics, SEV type, baseline patient and procedural characteristics, balloon dilatation techniques (PRE-BAV or POST-DIL), procedural outcomes, clinical outcomes, echocardiographic findings, safety endpoints, and any reported measures of valve malexpansion. Device success was defined according to the Valve Academic Research Consortium (VARC) criteria used in each study (26). As multiple versions of these definitions exist (VARC, VARC-2, and VARC-3) and differ across studies, device success outcomes were pooled only among studies using the same VARC standard.

### Risk of bias and quality assessment

The risk of bias was assessed using Version 2 of the Cochrane Risk-of-Bias tool (RoB2) for randomised studies (27), and the 9-point Newcastle-Ottawa Scale (NOS) for non-randomised studies (28). The RoB2 evaluates bias across five domains: (1) randomization process; (2) deviations from intended interventions; (3) missing data; (4) outcome measurement; (5) selection of the reported result, and provides an overall risk-of-bias rating (27). The NOS assesses bias across three domains, producing a total score ranging from 0 to 9. Studies were classified as poor quality (0–4), fair quality (5–6), or high quality (7–9) (29).

### Statistical analysis

A random-effects model meta-analysis was performed to estimate the pooled odds ratio (OR) and 95% confidence interval (CI) of all outcomes. The Hartung-Knapp-Sidik-Jonkman (HKSJ) method was used to account for uncertainty in between-study variance estimation and to provide more robust confidence interval estimation, particularly in meta-analyses involving relatively few studies (30). For sparse subgroup analyses with only two highly concordant studies and negligible heterogeneity, conventional random-effects models without HKSJ adjustment were used to avoid unstable confidence interval estimation.

Studies reporting an outcome with zero events in both treatment arms were retained in the forest plots to indicate that the outcome had been reported, but as they provided no information on the relative treatment effect, they contributed no information to the pooled odds ratio and therefore received no statistical weight. For studies with zero events in a single treatment arm, a continuity correction of 0.5 was applied. Where randomised data were included, stratified analyses were performed to distinguish between randomised and non-randomised studies, allowing for a subgroup analysis of high-quality randomised data alongside the overall analysis. The degree of statistical heterogeneity was quantified using the *I*-squared (*I*^2^) statistic, where *I*^2^ > 50% indicated moderate heterogeneity, and *I*^2^ > 75% indicated high heterogeneity (31). Publication bias was assessed for outcomes including ≥10 studies using funnel plots, Egger's regression, and Begg-Mazumdar tests. Outcomes with <10 studies were not assessed due to limited statistical power.

Baseline patient and procedural characteristics were presented descriptively for both intervention and control groups (PRE-BAV vs. DIRECT or POST-DIL vs. AS-DEPLOYED). Continuous variables are presented as mean ± standard deviation, and categorical variables as proportions (%). All statistical analyses were conducted using the “meta” package in R Statistical Software Version 4.4.2 (R Foundation for Statistical Computing, Vienna, Austria) (32).

## Results

**Figure 1:**
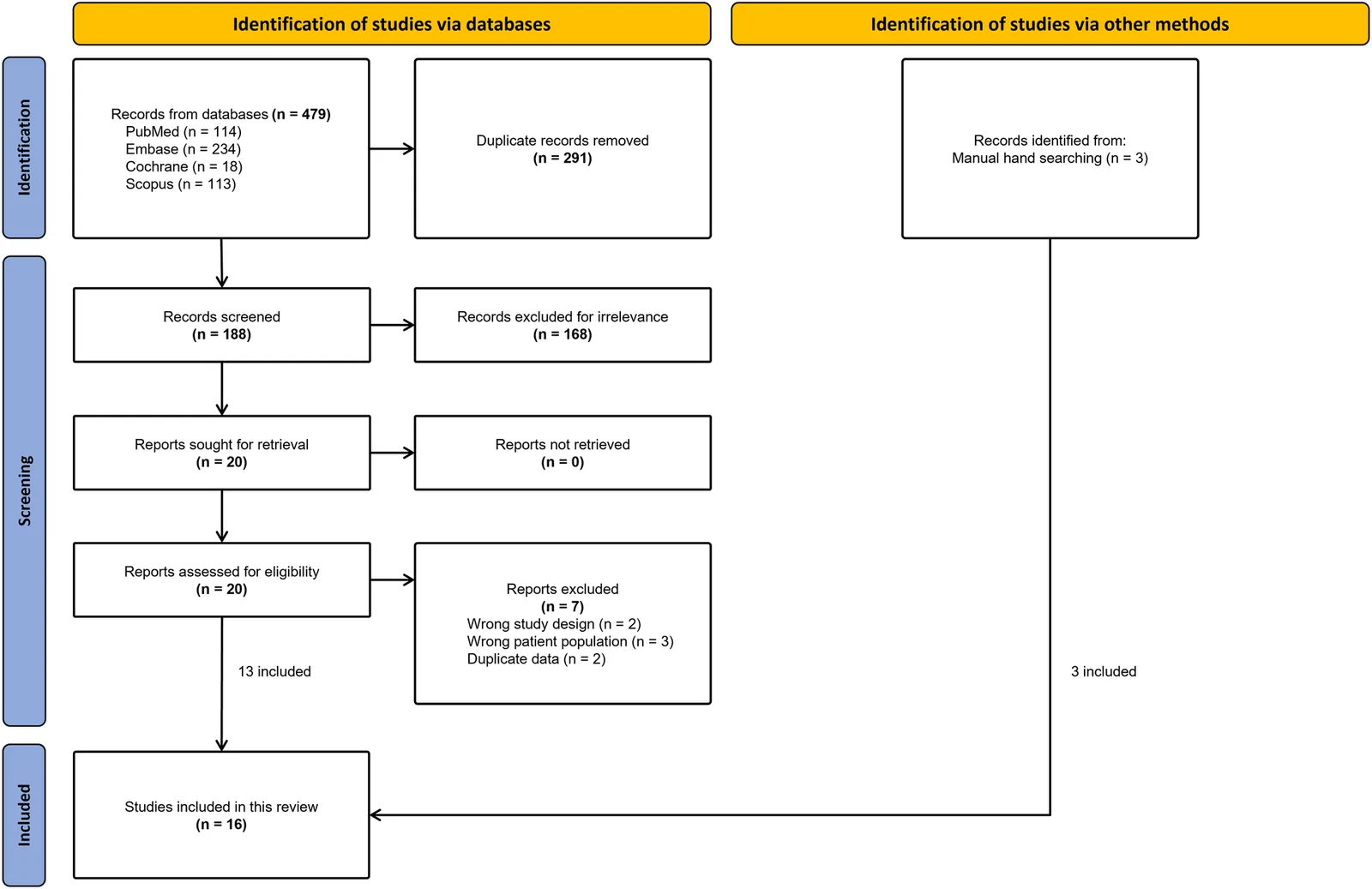
PRISMA flow diagram.

The search strategy identified 479 records across the different databases (Figure 1). After removing 291 duplicates, 188 unique records were screened for relevance. Following the exclusion of 168 records unrelated to balloon dilatation in SEV-TAVI, 20 full-text articles were retrieved and assessed for eligibility. Seven studies were excluded: two were non-full-text abstract publications (33, 34), three including predominantly patients receiving BEV-TAVI (35–37), and two for reporting duplicate data (38, 39). Three additional studies were identified through manual searching (7, 40, 41). The final analysis included 16 studies, comprising eight from Europe (7, 17, 42–47), two from the United States (2, 48), one from West Asia (49), and five international studies with multinational data (10, 40, 41, 50, 51). All studies were published between 2011 and 2024.

For PRE-BAV, eleven studies (nine observational, two randomised) with a total of 24,143 patients were analysed, of whom 17,420 (72.2%) underwent PRE-BAV (7, 10, 40–44, 48–51). For POST-DIL, five observational studies with 7,526 patients were included, with 1,909 (25.4%) undergoing POST-DIL (2, 17, 45–47). We identified no randomised studies of POST-DIL. Baseline study characteristics are summarized on Table 1. The risk of bias assessment for both randomised and non-randomised studies, according to RoB2 and NOS methodologies, is presented in Table 2. All studies were found to have an acceptable risk of bias. Publication bias was assessed for PRE-BAV outcomes including new PPM implantation, vascular complications, need for POST-DIL, and moderate or greater PVL (PVL2+), as these were the only outcomes reported by ≥10 studies. Visual inspection of the funnel plots, along with Egger's regression and Begg-Mazumdar tests showed no funnel plot asymmetry or statistically significant evidence of publication bias (Supplementary Figure S1 and Supplementary Table S3).

**Table 1:** Study characteristics.

Pre-dilatation balloon aortic valvuloplasty (PRE-BAV)											
No.	First author	Year	Country	Study design	Study period	Valve type	Total	PRE-BAV	DIRECT	DIRECT allocation	Follow-up
1.	Grube et al. (10)	2011	Germany, Brazil, Argentina, United Kingdom	Cohort study	2009–2010	CoreValve 100%	186	126	60	Operator discretion	30-day
2.	Fiorina et al. (42)	2014	Italy	Cohort study	2012–2013	CoreValve 100%	100	45	55	Excluded from no BAV if: Pure AR, degenerated surgical bioprosthesis, bicuspid AV, prior PRE-BAV, Need PRE-BAV for coronary protection	30-day
3.	Kochman et al. (43)	2014	Poland	Cohort study	2010–2013	CoreValve 100%	24	16	8	Sequential enrolment	30-day, 1-year
4.	Toutouzas et al. (40)	2016	Greece, Germany	Cohort study	2008–2013	CoreValve 100%	210	120	90	Sequential enrolment	30-day, 1-year
5.	Kim et al. (44)	2017	Germany	Cohort study	2014–2016	Acurate neo 100%	96	48	48	Operator discretion	30-day
6.	Rogers et al. (48)	2018	USA	Cohort study	2011–2012	CoreValve 100%	2,721	2,118	603	Operator discretion	30-day, 1-year
7.	Pagnesi et al. (50)	2020	Germany, Italy, Switzerland, Austria, United Kingdom, Brazil, Netherlands, Canada, Spain, Ireland	Cohort study	2012–2018	Acurate neo 100%	1,262	1,051	211	Operator discretion	30-day
8.	Benetos et al. (51)	2020	Greece, Israel	Randomised controlled trial	2015–2018	CoreValve 11.7%, Evolut-R 83.6%, Evolut-PRO 4.7%	171	86	85	Randomised	30-day, 1-year
9.	Alushi et al. (7)	2022	Germany	Cohort study	2011–2017	Self-expanding valves (unspecified)	19,066^a^	13,656	5,410	Operator discretion	30-day
10.	Arslan et al. (49)	2023	Turkey	Randomised controlled study	2019–2022	Evolut-R 100%	139	70	69	Sequential randomisation	30-day
11.	Elkoumy et al. (41)	2024	Germany, Sweden, Denmark, Austria, Netherlands	Cohort study	2020–2023	Acurate neo2 100%	168	84	84	Operator discretion	30-day

Balloon post-dilatation (POST-DIL)											
No.	First author	Year	Country	Study design	Study period	Valve type	Total	POST-DIL	AS-DEPLOYED	POST-DIL allocation	Follow-up
1.	Stundl et al. (45)	2016	Germany	Cohort study	2011–2013	CoreValve 100%	210	85	125	AR index <25, PVL 2+, Incomplete frame expansion	30-day, 1-year
2.	Harrison et al. (2)	2017	USA	Cohort study	2011–2012	CoreValve 100%	3,532	782	2,750	Residual gradients, PVL 2+, Incomplete frame expansion	30-day, 1-year
3.	Kim et al. (17)	2022	Germany	Cohort study	2012–2021	Acurate neo 100%	1,417	521	896	Residual gradients, PVL 2+, Incomplete frame expansion	30-day
4.	Massoullié et al. (46)	2023	France	Cohort study	2016–2019	CoreValve 100%	532	104	428	AR index <25, PVL 2+, Incomplete frame expansion	30-day, 1-year
5.	Sanz Sánchez (47)	2023	Italy	Cohort study	2007–2014	CoreValve & Evolut R	1,835	417	1,418	Operator discretion	30-day, 6-years

Summary of included studies evaluating PRE-BAV and POST-DIL in SEV-TAVI. The table lists the first author, year of publication, country of study origin, study design (randomised or observational), valve platform used, allocation criteria into the DIRECT or POST-DIL arms, and available follow-up duration. SEV, self-expanding valves.

a Data represent the SEV subgroup of the German Aortic Valve Registry (GARY) reported by Alushi et al.

**Table 2:** Quality/risk assessment.

PRE-BAV non-randomised studies—Newcastle-Ottawa Scale												
		Selection				Comparability		Outcome			Overall	
Study and year	Design	1	2	3	4	5	6	7	8	9	Score (*)	Quality
Grube et al. (10) (2011)	Prospective cohort study	*		*	*			*	*	*	6	Fair
Fiorina et al. (42) (2014)	Prospective cohort study	*	*	*	*			*	*	*	7	High
Kochman et al. (43) (2014)	Retrospective cohort study	*	*	*	*	*	*	*	*	*	9	High
Toutouzas et al. (40) (2016)	Retrospective cohort study	*	*	*	*			*	*	*	7	High
Kim et al. (44) (2017)	Retrospective cohort study	*	*	*	*	*	*	*	*	*	9	High
Rogers et al. (48) (2018)	Retrospective cohort study	*	*	*	*			*	*	*	7	High
Pagnesi et al. (50) (2020)	Retrospective cohort study	*	*	*	*			*	*	*	7	High
Alushi et al. (7) (2022)	Prospective cohort study	*	*	*	*	*	*	*	*	*	9	High
Elkoumy et al. (41) (2024)	Retrospective cohort study	*	*	*	*	*	*	*	*	*	9	High

PRE-BAV randomised studies—Cochrane Risk-of-Bias Tool 2												
		Domain 1	Domain 2	Domain 3	Domain 4	Domain 5						
Study and year	Design	Randomisation Process	Deviations from Intended Interventions	Missing Data	Measurement of the Outcome	Selection of the Reported Result	Overall risk					
Benetos et al. (51) (2020)	Randomised controlled trial	Low risk	Some concern	Low risk	Low risk	Low risk	Low					
Arslan et al. (49) (2023)	Randomised controlled study	Low risk	Some concern	Low risk	Low risk	Low risk	Low					

POST-DIL non-randomised studies—Newcastle-Ottawa Scale												
		Selection				Comparability		Outcome			Overall	
Study and year	Design	1	2	3	4	5	6	7	8	9	Score (*)	Quality
Stundl et al. (45) (2016)	Prospective cohort study	*	*	*	*			*	*	*	7	High
Harrison et al. (2) (2017)	Prospective cohort study	*	*	*	*			*	*	*	7	High
Kim et al. (17) (2022)	Retrospective cohort study	*	*	*	*			*	*	*	7	High
Massoullié et al. (46) (2023)	Retrospective cohort study	*	*	*	*			*	*	*	7	High
Sanz Sanchez et al. (47) (2023)	Prospective cohort study	*	*	*	*			*	*	*	7	High

Assessment of study quality and risk of bias for PRE-BAV and POST-DIL studies using the 9-point Newcastle–Ottawa Scale for observational studies (poor quality: 0–4 points; fair quality: 5–6 points; high quality: 7–9 points) and the Cochrane Risk of Bias 2 (RoB 2) tool for randomised studies.

### PRE-BAV analysis

#### Patient and procedural characteristics

Baseline patient and procedural characteristics for the PRE-BAV and DIRECT groups are presented in Tables 3, 4, except for Alushi et al., who did not report baseline characteristics separately for the SEV subgroup. Across included studies, mean patient age in PRE-BAV and DIRECT cohorts was reported in the early 80s, with study-level means ranging from 78.1 to 83.3 years. The proportion of male patients varied widely across studies, with reported study-level proportions ranging from 11.9% to 68.7% across PRE-BAV and DIRECT cohorts.

**Table 3:** Baseline patient characteristics.

PRE-BAV patient characteristics																									
No.	Study and year	Mean age (years ± SD)		Male (%)		Surgical risk score		Diabetes (%)		HTN (%)		CAD (%)		Prior CABG (%)		NYHA III/IV (%)		Prior CVA/TIA (%)		COPD (%)		CKD (%)		Mean LVEF (%±SD)	
		PRE-BAV	DIRECT	PRE-BAV	DIRECT	PRE-BAV	DIRECT	PRE-BAV	DIRECT	PRE-BAV	DIRECT	PRE-BAV	DIRECT	PRE-BAV	DIRECT	PRE-BAV	DIRECT	PRE-BAV	DIRECT	PRE-BAV	DIRECT	PRE-BAV	DIRECT	PRE-BAV	DIRECT
1.	Grube et al. (10) (2011)	81.9 ± 6.4	80.1 ± 6.4	42.9	46.7	ES 23.4	ES 23.3	26.2	18.3	79.4	41.7	65.9	61.7	26.2	23.3	74.6	86.7	22.2	3.3	23.0	18.3	43.7	26.7	–	–
2.	Fiorina et al. (42) (2014)	83.0 ± 8.0	83.0 ± 7.0	55.6	49.1	STS 7	STS 10	22.2	36.4	68.9	80.0	42.2	47.3	13.3	10.9	–	–	–	–	–	–	–	–	46.0 ± 17.0	49.0 ± 13.0
3.	Kochman et al. (43) (2014)	83.3 ± 3.7	78.1 ± 8.4	68.7	50.0	ES 18.6	ES 19.7	37.5	50.0	–	–	50.0	62.5	–	–	62.6	75.0	18.8	12.5	37.5	12.5	25.0	62.5	46.8 ± 5.0	38.4 ± 5.0
4.	Toutouzas et al. (40) (2016)	82.0 ± 6.0	79.0 ± 17.0	45.0	43.3	LES 19.0	LES 21.0	44.0	48.0	35.0	42.0	32.0	30.0	–	–	–	–	–	–	–	–	–	–	46.0 ± 8.0	50.0 ± 3.0
5.	Kim et al. (44) (2017)	82.2 ± 1.7	81.9 ± 2.0	33.3	29.2	STS 5.2	STS 4.4	45.8	39.6	33.3	27.1	62.5	56.3	16.7	10.4	–	–	–	–	20.8	33.3	–	–	64.5 ± 2.5	65.0 ± 2.2
6.	Rogers et al. (48) (2018)	83.3 ± 7.7	83.3 ± 7.7	57.5	50.2	STS 8.7	STS 8.5	38.0	34.7	–	–	37.6	47.1	–	–	85.5	87.7	12.2	16.1	–	–	–	–	53.8 ± 13.1	52.6 ± 16.1
7.	Pagnesi et al. (50) (2020)	81.9 ± 5.8	81.1 ± 5.8	35.6	32.7	STS 5.0	STS 5.2	–	–	87.4	90.9	40.9	41.2	11.8	10.9	77.1	81.5	9.2	15.4	18.5	23.7	–	–	57.0 ± 11.4	55.8 ± 13.7
8.	Benetos et al. (51) (2020)	82.1 ± 7.4	81.3 ± 7.0	47.7	57.6	LES II 25.8	LES II 23.0	30.2	35.3	–	–	40.7	50.6	–	–	86.0	83.5	–	–	23.3	22.4	24.4	14.1	51.3 ± 9.6	49.6 ± 10.0
9.	Alushi et al. (7) (2022)	–	–	–	–	–	–	–	–	–	–	–	–	–	–	–	–	–	–	–	–	–	–	–	–
10.	Arslan et al. (49) (2023)	78.8 ± 7.1	78.7 ± 7.3	52.9	43.5	STS 9.6	STS 9.5	37.1	39.1	77.1	73.9	–	–	12.9	8.7	–	–	–	–	22.8	17.4	48.6	42.0	53.3 ± 11.3	54.5 ± 10.0
11.	Elkoumy et al. (41) (2024)	82.0 ± 6.0	81.5 ± 6.9	11.9	48.8	LES II 3.5	LES II 2.8	30.0	38.0	82.0	89.0	32.0	36.0	3.2	4.8	56.0	69.0	12.0	13.0	26.0	12.0	–	–	53.5 ± 11.7	59.4 ± 11.4

POST-DIL Patient Characteristics																									
No.	Study and year	Mean age (Years ± SD)		Male (%)		Surgical risk score		Diabetes (%)		HTN (%)		CAD (%)		Prior CABG (%)		NYHA III/IV (%)		Prior CVA/TIA (%)		COPD (%)		CKD (%)		Mean LVEF (%±SD)	
		POST-DIL	A-D	POST-DIL	A-D	POST-DIL	A-D	POST-DIL	A-D	POST-DIL	A-D	POST-DIL	A-D	POST-DIL	A-D	POST-DIL	A-D	POST-DIL	A-D	POST-DIL	A-D	POST-DIL	A-D	POST-DIL	A-D
1.	Stundl et al. (45) (2016)	82.9 ± 6.3	80.6 ± 6.7	52.9	53.6	STS 7.3	STS 6.3	–	–	–	–	74.1	68.0	14.1	20.8	–	–	–	–	27.1	27.2	76.5	61.6	51.8 ± 13.3	51.1 ± 15.6
2.	Harrison et al. (2) (2017)	83.6 ± 7.9	83.3 ± 7.7	62.5	50.7	STS 8.5	STS 9.0	36.2	38.3	91.8	93.0	79.8	78.5	35.4	34.9	86.2	87.9	–	–	–	–	–	–	53.5 ± 13.8	54.1 ± 13.7
3.	Kim et al. (17) (2022)	81.7 ± 1.7	82.0 ± 1.6	40.5	31.6	ES II 2.9	ES II 3.1	–	–	–	–	56.2	57.8	–	–	–	–	–	–	–	–	–	–	65.0 ± 1.7	65.0 ± 1.3
4.	Massoullié et al. (46) (2023)	83.0 ± 6.0	82.0 ± 6.0	53.9	50.5	LES 13.6	LES 13.9	28.9	30.6	–	–	41.7	49.1	7.7	9.3	–	–	9.6	7.9	7.7	13.3	–	–	60.0 ± 11.0	59.0 ± 11.0
5.	Sanz Sanchez et al. (47) (2023)	81.0 ± 7.0	82.0 ± 6.0	72.0	56.6	STS 6.4	STS 5.9	27.3	31.2	81.1	81.1	42.1	44.1	17.0	13.9	82.4	75.0	16.4	14.4	20.1	22.4	22.1	21.4	49.8 ± 13.4	51.6 ± 12.2

Summary of baseline demographic and clinical characteristics for patients undergoing PRE-BAV and POST-DIL in SEV-TAVI studies. A-D, AS-DEPLOYED; ES, EuroSCORE; STS, Society of Thoracic Surgeons Predicted Risk of Mortality Score; LES II, Logistic EuroSCORE II; ES II, EuroSCORE II; HTN, hypertension; CAD, coronary artery disease; CABG, coronary artery bypass grafting; NYHA, New York Heart Association; CVA/TIA, cerebrovascular accident/transient ischemic attack; COPD, chronic obstructive pulmonary disease; CKD, chronic kidney disease; LVEF, left ventricular ejection fraction.

**Table 4:** Baseline procedural characteristics.

PRE-BAV procedure characteristics																									
No.	Study and year	Trans-femoral access (%)		Trans-subclavian access (%)		Trans-aorta access (%)		Mean aortic valve gradient (mmHg)		Mean Aortic valve area (cm^2^)		Aortic annulus diameter (mm)		Acurate neo 23 mm (%)		Acurate neo 25 mm (%)		CoreValve/Evolut 26 mm (%)		Acurate neo 27 mm (%)		CoreValve/Evolut 29 mm (%)		CoreValve/Evolut ≥31 mm (%)	
		PRE-BAV	DIRECT	PRE-BAV	DIRECT	PRE-BAV	DIRECT	PRE-BAV	DIRECT	PRE-BAV	DIRECT	PRE-BAV	DIRECT	PRE-BAV	DIRECT	PRE-BAV	DIRECT	PRE-BAV	DIRECT	PRE-BAV	DIRECT	PRE-BAV	DIRECT	PRE-BAV	DIRECT
1.	Grube et al. (10) (2011)	–	–	–	–	–	–	46.8 ± 13.0	47.8 ± 4.0	0.72 ± 0.11	0.66 ± 0.12	–	–	–	–	–	–	–	–	–	–	–	–	–	–
2.	Fiorina et al. (42) (2014)	71.1	61.8	8.9	16.4	20.0	21.8	48.0 ± 16.0	44.0 ± 13.0	0.36 ± 0.10	0.39 ± 0.10	–	–	–	–	–	–	20.0	16.4	–	–	40.0	60.0	40.0	24.6
3.	Kochman et al. (43) (2014)	81.3	62.5	18.7	37.5	0.0	0.0	55.9 ± 12.0	46.0 ± 14.1	0.59 ± 0.17	0.58 ± 0.15	–	–	–	–	–	–	25.0	25.0	–	–	37.5	37.5	37.5	37.5
4.	Toutouzas et al. (40) (2016)	92.0	92.0	8.0	7.0	0.0	1.0	43.0 ± 15.0	49.0 ± 14.0	0.60 ± 0.20	0.60 ± 0.10	24.0 ± 3.0	20.0 ± 5.0	–	–	–	–	46.7	41.1	–	–	48.3	48.9	3.3	6.7
5.	Kim et al. (44) (2017)	–	–	–	–	–	–	35.5 ± 3.9	38.5 ± 5.1	0.80 ± 0.07	0.80 ± 0.07	23.0 ± 0.5	22.4 ± 0.6	29.2	41.7	54.2	41.7	–	–	16.7	16.7	–	–	–	–
6.	Rogers et al. (48) (2018)	81.5	80.6	4.7	5.3	13.7	14.1	47.7 ± 12.5	45.4 ± 13.5	0.67 ± 0.30	0.69 ± 0.29	–	–	–	–	–	–	19.8	26.0	–	–	42.6	35.3	36.8	36.0
7.	Pagnesi et al. (50) (2020)	100.0	100.0	0.0	0.0	0.0	0.0	45.4 ± 16.6	34.0 ± 13.6	0.69 ± 0.19	0.76 ± 0.17	26.3 ± 2.1	25.7 ± 2.4	26.3	34.1	42.5	34.6	–	–	31.2	31.3	–	–	–	–
8.	Benetos et al. (51) (2020)	96.5	96.5	3.5	3.5	0.0	0.0	49.4 ± 14.8	47.2 ± 15.0	0.65 ± 0.15	0.69 ± 0.16	23.7 ± 3.1	23.1 ± 2.4	–	–	–	–	–	–	–	–	–	–	–	–
9.	Alushi et al. (7) (2022)	–	–	–	–	–	–	–	–	–	–	–	–	–	–	–	–	–	–	–	–	–	–	–	–
10.	Arslan et al. (49) (2023)	100.0	100.0	0.0	0.0	0.0	0.0	54.0 ± 11.3	52.6 ± 11.1	0.71 ± 0.15	0.72 ± 0.15	25.6 ± 2.7	25.4 ± 2.7	–	–	–	–	18.6	15.9	–	–	32.9	42.0	44.3	34.8
11.	Elkoumy et al. (41) (2024)	100.0	100.0	0.0	0.0	0.0	0.0	38.5 ± 10.9	33.5 ± 14.5	0.80 ± 0.15	0.80 ± 0.15	22.4 ± 1.9	22.6 ± 1.8	35.7	34.5	45.2	41.7	–	–	19.1	23.8	–	–	–	–

POST-DIL procedure characteristics																									
No.	Study and year	Trans-femoral access (%)		Trans-subclavian access (%)		Trans-aorta access (%)		Mean aortic valve gradient (mmHg)		Aortic valve area (cm^2^)		Aortic annulus diameter (mm)		Acurate neo 23 mm (%)		Acurate neo 25 mm (%)		CoreValve/Evolut 26 mm (%)		Acurate neo 27 mm (%)		CoreValve/Evolut 29 mm (%)		CoreValve/Evolut ≥31 mm (%)	
		POST-DIL	A-D	POST-DIL	A-D	POST-DIL	A-D	POST-DIL	A-D	POST-DIL	A-D	POST-DIL	A-D	POST-DIL	A-D	POST-DIL	A-D	POST-DIL	A-D	POST-DIL	A-D	POST-DIL	A-D	POST-DIL	A-D
1.	Stundl et al. (45) (2016)	97.6	96.0	1.2	0.8	1.2	3.2	46.7 ± 17.2	39.7 ± 15.4	0.67 ± 0.17	0.74 ± 0.18	23.9 ± 2.6	23.4 ± 2.6	–	–	–	–	24.7	29.6	–	–	43.5	39.2	23.5	24.0
2.	Harrison et al. (2) (2017)	–	–	–	–	–	–	44.8 ± 16.5	41.3 ± 15.7	–	–	25.2 ± 2.2	24.4 ± 2.2	–	–	–	–	17.9	27.5	–	–	42.1	46.0	38.1	24.9
3.	Kim et al. (17) (2022)	100.0	100.0	0.0	0.0	0.0	0.0	45.0 ± 4.3	39.0 ± 4.9	0.70 ± 0.08	0.70 ± 0.05	24.0 ± 0.6	23.6 ± 0.6	19.0	28.2	47.6	39.4	–	–	33.4	32.4	–	–	–	–
4.	Massoullié et al. (46) (2023)	76.0	64.0	24.0	36.0	0.0	0.0	55.0 ± 18.0	43.0 ± 13.0	0.64 ± 0.22	0.74 ± 0.22	23.8 ± 2.1	23.8 ± 2.4	–	–	–	–	38.5	33.4	–	–	49.0	47.4	8.7	12.6
5.	Sanz Sanchez et al. (47) (2023)	83.2	80.8	11.1	13.6	5.8	5.2	53.8 ± 14.4	51.4 ± 14.9	–	–	–	–	–	–	–	–	34.3	49.5	–	–	50.6	42.3	12.9	6.3

Overview of procedural details for PRE-BAV and POST-DIL groups, including access route, pre-procedural echocardiographic baseline parameters, along with valve platform and valve size used. A-D, AS-DEPLOYED.

Reported surgical risk varied substantially across studies, reflecting the use of different risk scoring systems, including EuroSCORE (ES), EuroSCORE II (ES II), logistic EuroSCORE (LES), logistic EuroSCORE II (LES II), and the Society of Thoracic Surgeons (STS) score. The prevalence of cardiovascular comorbidities (hypertension, coronary artery disease, chronic kidney disease, and prior cerebrovascular events) varied across studies, with some reporting numerically higher rates in PRE-BAV cohorts and others showing similar distributions between the two groups. Functional status, where reported, indicated a high prevalence (>50%) of NYHA class III/IV symptoms in both PRE-BAV and DIRECT groups across most studies.

Echocardiographic characteristics, including left ventricular ejection fraction (LVEF) and baseline aortic valve parameters, were broadly similar within individual studies, although reported values varied across studies. Mean aortic valve area, aortic valve gradients, and annular diameters showed overlapping ranges between PRE-BAV and DIRECT cohorts across different studies. Transfemoral access was the predominant approach in both PRE-BAV and DIRECT groups, with limited use of alternative access routes reported in a minority of studies. Prosthesis sizing varied across different studies and between treatment groups within individual studies.

#### Procedural and device outcomes

**Figure 2:**
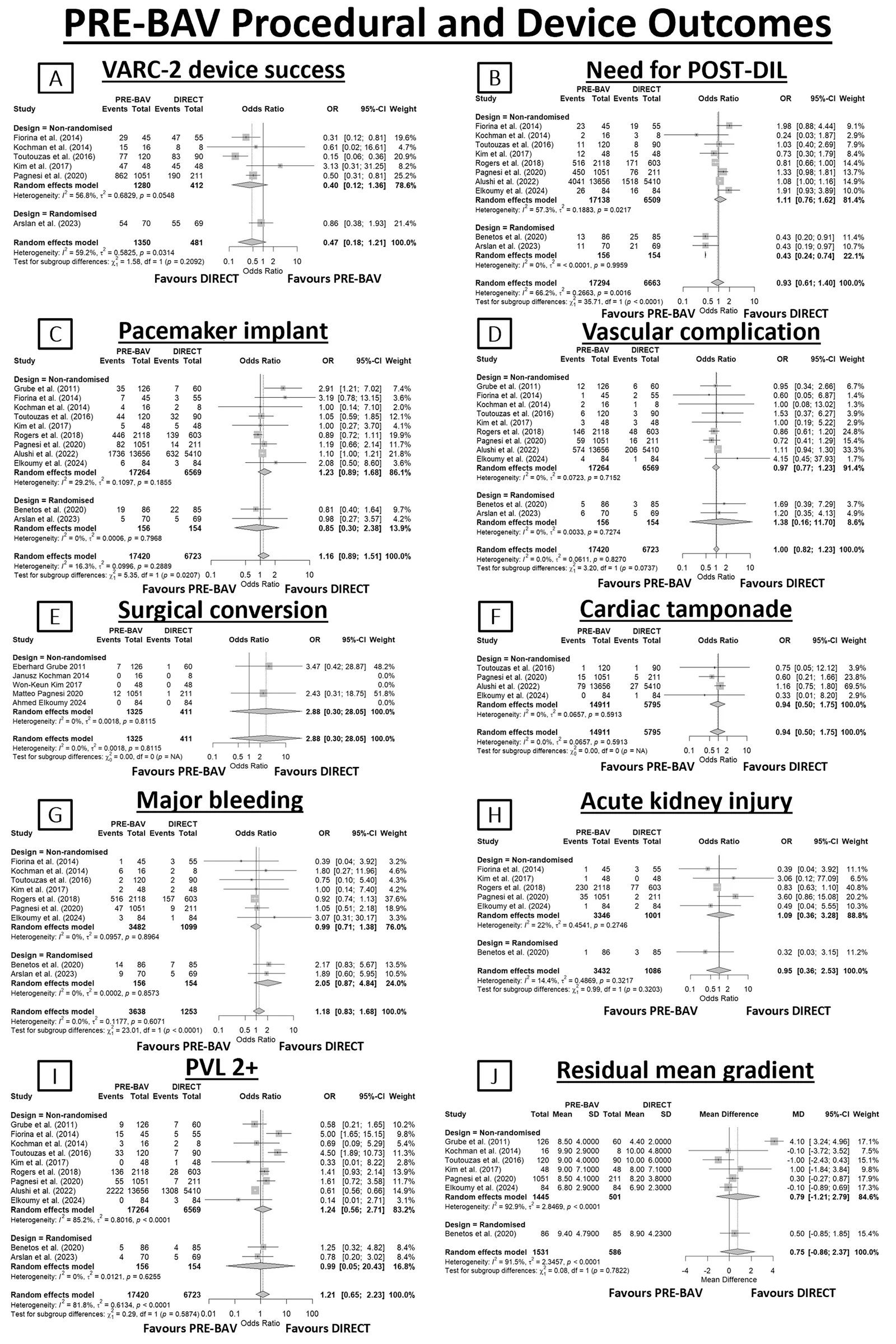
Procedural and device outcomes comparing PRE-BAV and DIRECT. Forest plots present odds ratios (ORs) with 95% confidence intervals for key procedural endpoints: (**A**) VARC-2-defined device success, (**B**) need for post-dilatation (POST-DIL), (**C**) permanent pacemaker implantation, (**D**) vascular complication, (**E**) surgical conversion, (**F**) cardiac tamponade, (**G**) major bleeding, (**H**) acute kidney injury, (**I**) moderate or greater paravalvular leak (PVL 2+), and (**J**) residual mean gradient. For positive outcomes like VARC-2 device success, an OR < 1 indicates worse performance for PRE-BAV relative to DIRECT.

Procedural and device outcomes for the PRE-BAV analysis are summarized in Figure 2. All included PRE-BAV studies reported data on new PPM implantation, vascular complications, and PVL2+. Reporting of other procedural and device outcomes was variable: VARC-2 device success (six studies) (40, 42–44, 49, 50), VARC-3 device success (one study; therefore precluding meta-analysis for this outcome) (41), need for post-dilatation (ten studies), surgical conversion (five studies), cardiac tamponade (four studies), major bleeding (nine studies), acute kidney injury (six studies), and residual mean gradient (seven studies). Across all endpoints, there were no statistically significant differences between PRE-BAV and DIRECT, including key outcomes such as new PPM implantation (OR 1.16; 95% CI 0.89–1.51) and the need for POST-DIL (OR 0.93; 95% CI 0.61–1.40). Surgical conversion occurred infrequently, resulting in sparse data and an imprecise pooled estimate. However, in a subgroup analysis restricted to randomised PRE-BAV studies, PRE-BAV was associated with a significantly lower need for POST-DIL compared with DIRECT (OR 0.43; 95% CI 0.24–0.74).

#### Clinical outcomes

**Figure 3:**
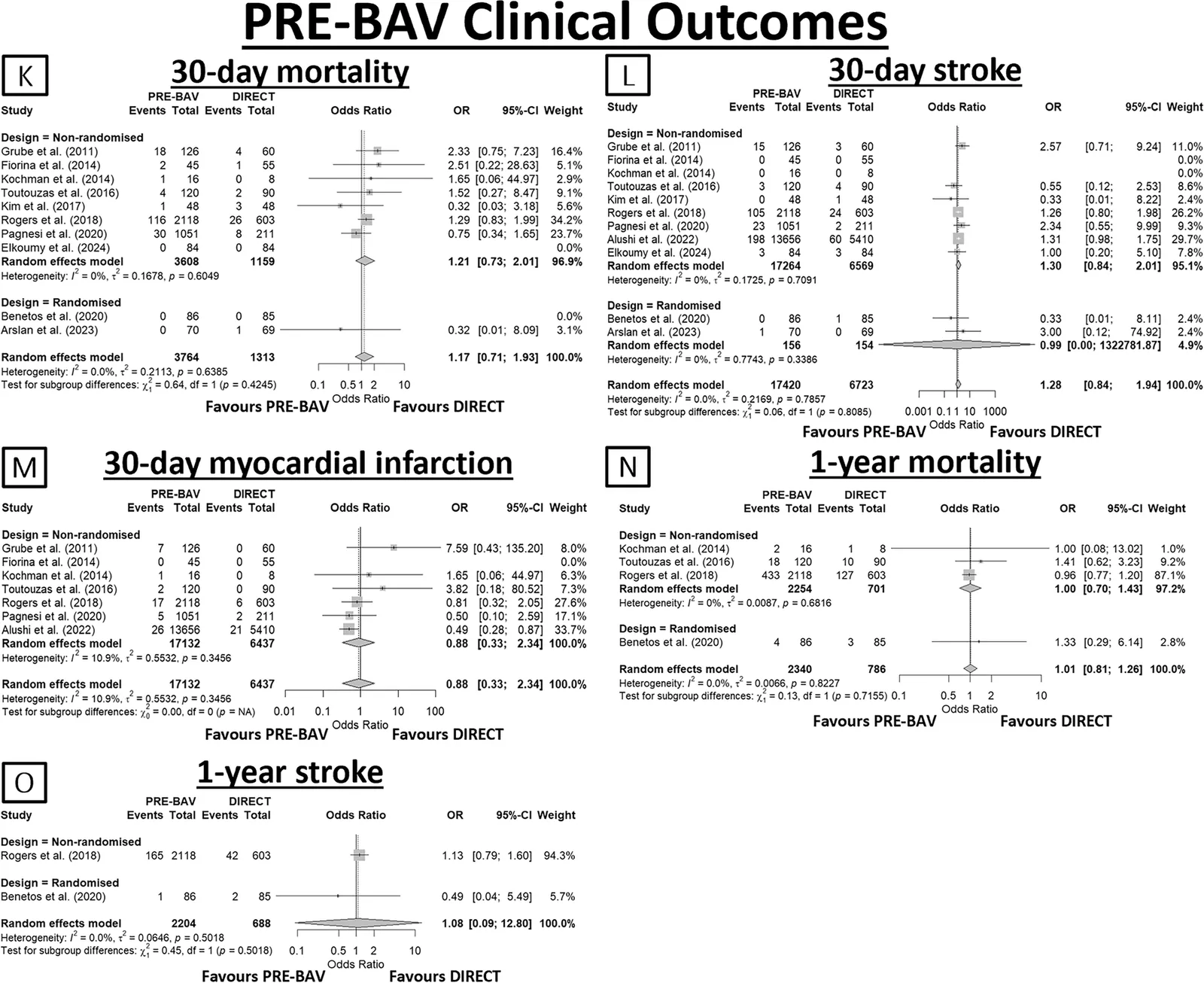
Clinical outcomes comparing PRE-BAV and DIRECT. Forest plots present odds ratios (ORs) with 95% confidence intervals for: (**K**) 30-day mortality, (**L**) 30-day stroke, (**M**) 30-day myocardial infarction, (**N**) 1-year mortality, and (**O**) 1-year stroke.

Clinical outcomes for the PRE-BAV analysis are summarized in Figure 3. Short-term outcomes were consistently reported, with all PRE-BAV studies reporting 30-day stroke, ten studies reporting 30-day mortality, and seven studies reporting 30-day myocardial infarction. In contrast, longer-term outcomes were less frequently reported, with only four studies reporting 1-year mortality and two studies reporting 1-year stroke. Across all clinical endpoints, there were no statistically significant differences between PRE-BAV and DIRECT at 30 days, including mortality (OR 1.17; 95% CI 0.71–1.93), stroke (OR 1.28; 95% CI 0.84–1.94), and myocardial infarction (OR 0.88; 95% CI 0.33–2.34). Similarly, no significant between-group differences were observed at 1 year for mortality or stroke.

### POST-DIL analysis

#### Patient and procedural characteristics

Baseline patient and procedural characteristics for POST-DIL and AS-DEPLOYED are summarized in Tables 3, 4. Across included studies, mean patient age in POST-DIL and AS-DEPLOYED cohorts was reported in the early 80s, with study-level means ranging from 81.0 to 83.6 years. The proportion of male patients varied across studies, with reported study-level proportions ranging from 40.5% to 72.0% across POST-DIL and AS-DEPLOYED cohorts.

As with PRE-BAV, reported surgical risk varied substantially across studies, using different risk scoring systems, including the LES, ES II, and STS score. The prevalence of cardiovascular comorbidities, including hypertension, coronary artery disease, chronic kidney disease, diabetes, and prior cerebrovascular events, varied across studies, with some reporting numerically higher rates in POST-DIL cohorts and others showing similar distributions between both groups. Functional status, where reported, also indicated a high prevalence (>50%) of NYHA class III/IV symptoms in both groups across the studies.

Echocardiographic characteristics, including LVEF and baseline aortic valve parameters, were generally similar within individual studies, although values varied across different studies. Mean aortic valve area, aortic valve gradients, and annular diameters showed overlapping ranges between both cohorts across different studies. Transfemoral access was the predominant procedural approach in both POST-DIL and AS-DEPLOYED groups, with alternative access routes used infrequently. Prosthesis sizing varied across studies and between treatment groups within individual studies.

#### Procedural and device outcomes

**Figure 4:**
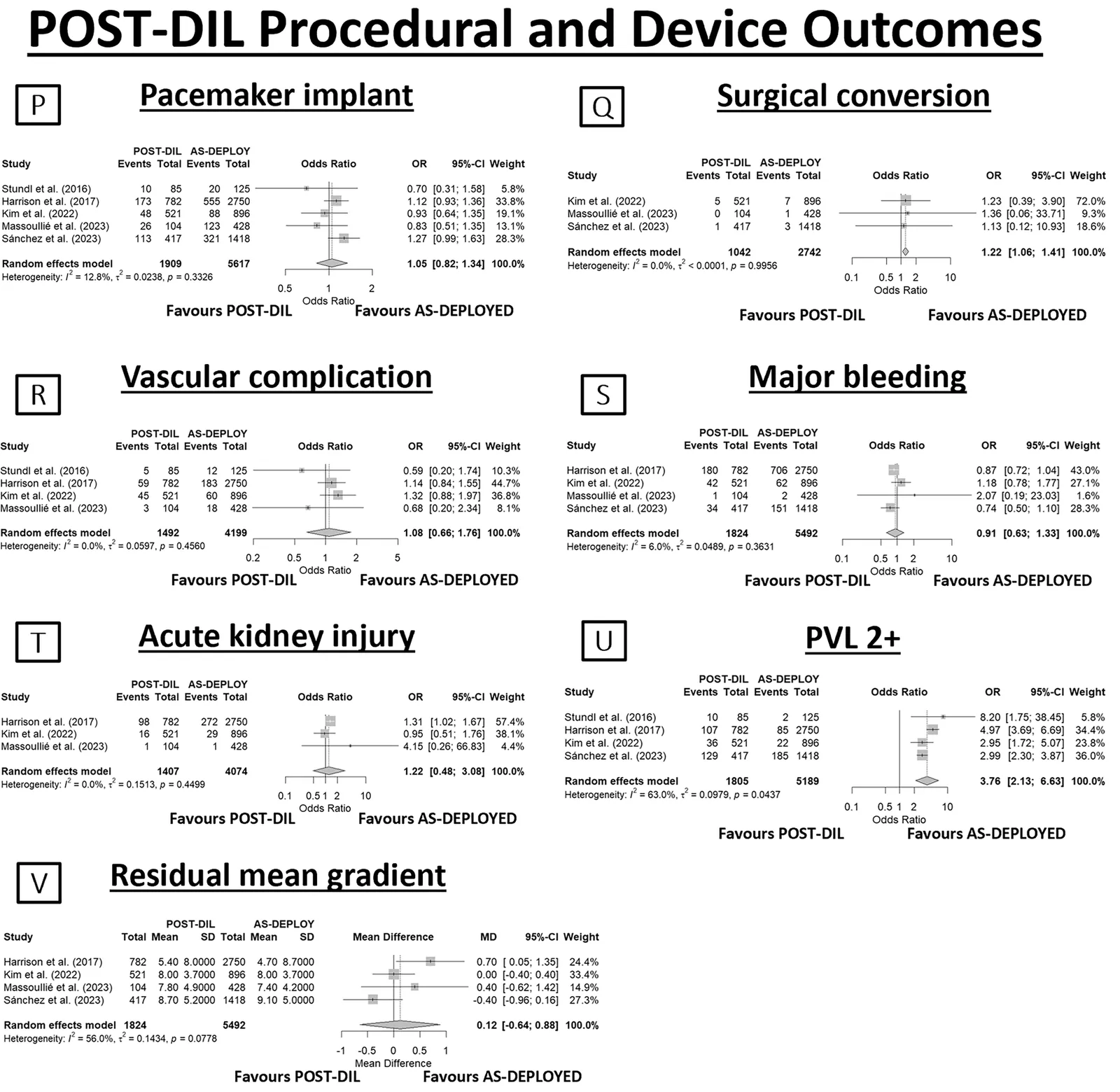
Procedural and device outcomes comparing POST-DIL and AS-DEPLOYED. Forest plots present odds ratios (ORs) with 95% confidence intervals for key procedural endpoints: (**P**) permanent pacemaker implantation, (**Q**) surgical conversion, (**R**) vascular complication, (**S**) major bleeding, (**T**) acute kidney injury, (**U**) moderate or greater paravalvular leak (PVL 2+), and (**V**) residual mean gradient.

Procedural and device outcomes for the POST-DIL analysis are summarized in Figure 4. All included POST-DIL studies reported data on new PPM implantation. Only one study reported VARC-2 device success, preventing meaningful meta-analysis for this outcome. Reporting of other procedural and device outcomes were variable: surgical conversion (three studies), vascular complications (four studies), major bleeding (four studies), acute kidney injury (three studies), PVL2+ (four studies), and residual mean gradient (four studies). As the requirement for POST-DIL was the exposure of interest, it was not reported as an outcome in any included study. Compared to the AS-DEPLOYED cohort, POST-DIL was associated with higher observed rates of surgical conversion (OR 1.22; 95% CI 1.06–1.41) and PVL 2+ (OR 3.76; 95% CI 2.13–6.63). No significant differences were observed for other endpoints, including new PPM implantation (OR 1.05; 95% CI 0.82–1.34). Two studies (Harrison et al. and Kim et al.) reported PVL severity after initial valve deployment and after POST-DIL. PVL2+ decreased from 58.1% to 14.2% in Harrison et al. and from 32.0% to 7.5% in Kim et al.

#### Clinical outcomes

**Figure 5:**
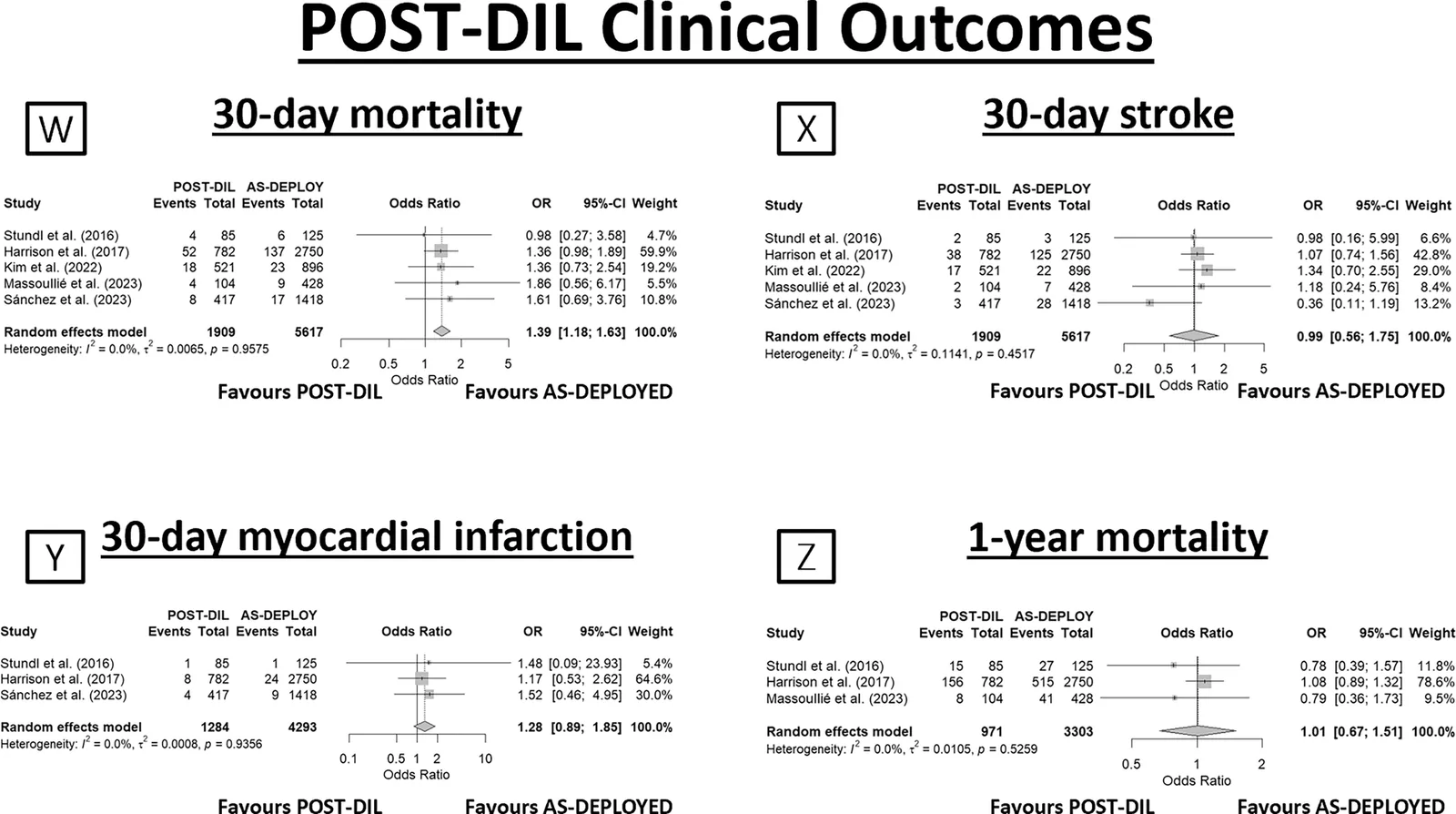
Clinical outcomes comparing POST-DIL and AS-DEPLOYED. Forest plots present odds ratios (ORs) with 95% confidence intervals for: (**W**) 30-day mortality, (**X**) 30-day stroke, (**Y**) 30-day myocardial infarction, and (**Z**) 1-year mortality.

Clinical outcomes for the POST-DIL analysis are summarized in Figure 5. Short-term outcomes were consistently reported across included studies, with all POST-DIL studies reporting 30-day mortality and stroke, while three studies reported 30-day myocardial infarction. Reporting of longer-term outcomes was limited, with only three studies reporting 1-year mortality and only one study reporting 1-year stroke, precluding meta-analysis of 1-year stroke. Compared to the AS-DEPLOYED cohort, POST-DIL was associated with higher observed 30-day mortality (OR 1.39; 95% CI 1.18–1.63). No significant differences were observed between groups for other outcomes, including 30-day stroke (OR 0.99; 95% CI 0.56–1.75), 30-day myocardial infarction (OR 1.28; 95% CI 0.89–1.85), and 1-year mortality (OR 1.01; 95% CI 0.67–1.57).

## Discussion

This study summarizes available evidence regarding the outcomes of both PRE-BAV and POST-DIL in patients undergoing SEV-TAVI. The main findings of this analysis, which included 11 PRE-BAV studies (24,143 patients) and 5 POST-DIL studies (7,526 patients), are as follows: (1) PRE-BAV was not associated with worse clinical or procedural outcomes, but in the subset of randomised studies, it was linked to a reduced need for POST-DIL; and (2) POST-DIL, when compared to the AS-DEPLOYED group, had higher observed rates of 30-day mortality, PVL2+, and conversion to surgery. Importantly, these associations should be interpreted in the context of procedural indication, as POST-DIL is not randomly assigned but performed in response to suboptimal valve deployment.

### Impact of PRE-BAV

PRE-BAV was initially routinely performed to fracture calcium deposits in the aortic valve complex, aiding the passage of the delivery system through the calcified native valve (52). The development of lower-profile delivery systems, improving operator experience, and streamlined procedures contributed to the decline in PRE-BAV use (39, 53–55).

Earlier meta-analyses comparing PRE-BAV and DIRECT were conducted before any randomised data for this comparison were available, and pooled data from both BEVs and SEVs without stratifying outcomes by valve type (56–58). These analyses reported inconsistent results for key clinical endpoints such as 30-day mortality, 30-day stroke, new PPM implantation, PVL2+, and the need for POST-DIL, with some reporting no differences, while others identifying significant effects.

By restricting our analysis to SEVs and incorporating two recent randomised studies (49, 51), our meta-analysis was able to add new insights through stratified analysis of randomised data. In the randomised studies, PRE-BAV was associated with a nearly 60% reduction in the need for POST-DIL (OR 0.43; 95% CI 0.24–0.74). This finding may be clinically relevant in contemporary SEV-TAVI practice, where high THV implantation, often facilitated by cusp-overlap deployment, is used to minimize conduction disturbances, while operators aim to avoid POST-DIL because of concerns regarding valve embolization (59, 60). Concurrently, advances in CT-guided valve sizing and newer-generation SEV platforms have also improved initial valve deployment and expansion, which may further reduce the need for further adjunctive balloon dilatation (60–62). Notably, this effect was observed only in randomised studies, supporting the possibility that PRE-BAV may facilitate more optimal valve expansion, thereby reducing the need for adjunctive intervention. In contrast, the absence of a similar signal in observational data highlights the impact of selection bias and unmeasured confounding inherent to non-randomised designs (63). However, these subgroup findings should be interpreted cautiously, as they are based on only two randomised studies with a modest sample size.

### Impact of POST-DIL

TAVI outcomes are influenced by native valve calcification (64), valve platform (65), and operator experience (66). Extensive aortic root calcification is associated with increased PVL (13, 67) and hence raises the likelihood of requiring POST-DIL (13, 68). POST-DIL is intended to improve valve apposition and reduce PVL (2, 47, 69–71) and is therefore performed during TAVI when initial valve deployment is deemed suboptimal. In all studies included in our meta-analysis, the decision to perform POST-DIL was made after THV deployment based on operator assessment using criteria such as PVL2+, incomplete frame expansion, residual transvalvular gradients, and aortic regurgitation index <25 (Table 1). As such, POST-DIL is inherently a response to procedural complications or suboptimal outcomes, and therefore identifies a higher-risk subgroup rather than representing an independent intervention applied across comparable patients (47).

In our pooled analysis, POST-DIL was associated with higher rates of PVL2+, surgical conversion, and 30-day mortality. These findings are most plausibly explained by confounding by indication and reverse causality, whereby POST-DIL is performed in response to adverse anatomical and procedural features rather than causing them. While only two studies reported PVL severity after initial valve deployment and after POST-DIL, both demonstrated reductions in PVL2+ following POST-DIL. However, residual PVL2+ persisted in a proportion of POST-DIL patients and remained more frequent compared to the AS-DEPLOYED cohort, suggesting that while POST-DIL may reduce PVL, it does not consistently eliminate clinically significant residual regurgitation.

Patients undergoing POST-DIL therefore likely represent cases with more complex anatomy, including greater calcific burden, eccentric calcification, challenging annular geometry, bicuspid anatomy, suboptimal implantation depth, or prosthesis underexpansion, all of which may independently contribute to worse outcomes (72–75). Persistence of residual PVL2+ or the need for surgical conversion despite POST-DIL likely reflects incomplete correction of the underlying procedural issue rather than harm caused by POST-DIL itself. While POST-DIL may rarely contribute directly to complications such as annular rupture, myocardial injury, or THV embolization, these mechanisms may not be the dominant drivers of the overall findings. Therefore, the associations between POST-DIL and adverse outcomes in observational series should not be interpreted as causal (63).

Our findings are consistent with a previous meta-analysis of observational studies comparing POST-DIL and AS-DEPLOYED in BEVs and SEVs, which reported greater PVL2+ and 30-day mortality with POST-DIL (mortality not reaching statistical significance) (76). After restricting to SEVs and adding three recent SEV-specific studies (2, 17, 46), the pattern remained, reinforcing the need to interrogate underlying determinants rather than attributing causality to POST-DIL.

Importantly, interpretation of longer-term outcomes remains limited. Only three POST-DIL studies reported 1-year mortality, and just one study reported 1-year stroke, precluding robust assessment of longer-term clinical impact. This limited follow-up perpetuates uncertainty regarding whether POST-DIL effectively mitigates THV malexpansion in a manner that translates into sustained clinical benefit.

### Addressing frame malexpansion

There is growing recognition that THV malexpansion adversely impacts outcomes, as demonstrated in the ACURATE IDE trial (20, 21). The DOUBLE-TAP study further showed that routine double POST-DIL can improve valve expansion, increase THV cross-sectional area, and reduce frame pin-wheeling, highlighting the potential effectiveness of contemporary balloon dilatation strategies in addressing malexpansion (77).

In this context, our findings suggest that selective PRE-BAV may facilitate more optimal THV expansion and reduce the need for POST-DIL in certain procedural scenarios, although the limited randomised evidence precludes definitive recommendations regarding routine use. This is particularly relevant in SEV-TAVI, where contemporary strategies favour higher implantation to reduce rates of PPM implantation (78–80), but consequently limit the procedural safety margin for POST-DIL due to the risk of valve “pop-up” or embolization (16, 60), thereby reinforcing that corrective strategies such as POST-DIL are typically reserved for more complex procedural scenarios.

Future research should focus on developing anatomy-specific PRE-BAV and POST-DIL protocols (balloon size to annulus, inflation pressure, staged or single dilatation), while incorporating core-lab adjudicated data to better assess the impact of balloon dilatation on THV malexpansion. Moreover, longer-term, adequately powered studies with standardized outcome reporting are needed to clarify the durability and clinical relevance of balloon dilatation strategies. Ultimately, randomised studies will be required to objectively define the optimal role of balloon dilatation techniques in contemporary TAVI practice.

### Limitations

This study has several limitations. First, the predominance of observational studies, particularly for the POST-DIL analysis where no randomised data were available, limits causal inference. The association between POST-DIL and adverse outcomes is highly susceptible to confounding by indication and reverse causality, as POST-DIL is performed in response to suboptimal procedural results. Residual PVL2+ is also a common indication for POST-DIL and an outcome of interest. Although two studies reported PVL2+ after initial valve deployment and after POST-DIL, only one included comparable AS-DEPLOYED data, preventing pooled quantitative analysis of PVL reduction. The effect of POST-DIL on PVL reduction could therefore only be described qualitatively. Second, there was substantial clinical and methodological heterogeneity across studies, including differences in patient selection, baseline risk profiles, procedural techniques, surgical risk scoring systems, and SEV platforms, encompassing both earlier-generation and contemporary devices. In addition, important anatomical variables such as annular calcification burden, calcium distribution, LVOT anatomy, and CT-derived sizing parameters were inconsistently reported and could not be analysed. Although conservative random-effects models with HKSJ adjustment were used to account for between-study variability, residual heterogeneity remains likely. Third, although randomised data were included in the PRE-BAV subgroup analysis to improve reliability, the limited number of randomised studies (*n* = 2) limits the robustness of these findings and raises the possibility of imprecise effect estimates. Fourth, this study-level meta-analysis could not adjust for baseline imbalances between exposure groups, including differences in patient characteristics and prosthesis selection, which may have contributed to residual confounding. Finally, inconsistent reporting of longer-term outcomes (1-year mortality and stroke) and key procedural endpoints (VARC-defined device success), together with the small number of studies available for several analyses, limited statistical power and generalizability to contemporary TAVI practice. A formal GRADE assessment was not performed, however, the major factors affecting the certainty of the evidence have been discussed throughout the manuscript.

## Conclusion

This meta-analysis included eleven studies on PRE-BAV (24,143 patients) and five observational studies on POST-DIL (7,526 patients) in SEV-TAVI, and reports that PRE-BAV was not associated with worse clinical or procedural outcomes, and in randomised data, significantly reduced the need for POST-DIL. In contrast, observational data suggest that POST-DIL had higher observed rates of 30-day mortality, surgical conversion, and PVL2+, although these findings are most likely explained by confounding by indication and should not be interpreted as causal. Further prospective randomised studies are required to define the optimal role and timing of balloon dilatation strategies in TAVI.

## Data Availability

The original contributions presented in the study are included in the article/Supplementary Material, further inquiries can be directed to the corresponding author/s.
