# Supplementary material for: Pre- and post-dilatation in transcatheter aortic valve implantation with self-expanding valves: a systematic review and meta-analysis
Source: Front Cardiovasc Med. 2026 Aug 26;13:1883882. doi: 10.3389/fcvm.2026.1883882 (PMC13553858; doi:10.3389/fcvm.2026.1883882)
Supplement: Supplementary file 1 [file Supplementaryfile1.docx]

Supplementary Table S1: PRISMA Checklist.

Supplementary Table S2: Full electronic search strategy for each database (search date: 18 November 2024).

| **Database** | **Search strategy** | **Records retrieved** |
| --- | --- | --- |
| **PubMed** | (((Aortic valve stenosis OR aortic stenosis) AND (Transcatheter aortic valve replacement OR TAVR OR transcatheter aortic valve implantation OR TAVI)) AND (CoreValve OR Evolut OR Evolut R OR Evolut PRO OR Evolut PRO+ OR ACURATE neo OR ACURATE neo2 OR Portico OR Allegra OR self-expanding valve OR self-expanding supraannular valve)) AND (Pre dilatation OR pre-dilatation OR pre dilation OR pre-dilation OR post dilatation OR post-dilatation OR post dilation OR post-dilation OR malexpansion OR incomplete expansion OR underexpansion OR suboptimal valve expansion OR frame deformation OR distorted valve frame OR non-uniform deployment OR irregular radial expansion) | 114 |
| **Embase** | ('aortic valve stenosis'/exp OR 'aortic valve stenosis' OR 'aortic stenosis'/exp OR 'aortic stenosis') AND ('transcatheter aortic valve replacement'/exp OR 'transcatheter aortic valve replacement' OR tavr OR 'transcatheter aortic valve implantation'/exp OR 'transcatheter aortic valve implantation' OR 'tavi'/exp OR tavi) AND ('corevalve'/exp OR 'corevalve' OR 'evolut'/exp OR 'evolut' OR 'evolut r'/exp OR 'evolut r' OR 'evolut pro'/exp OR 'evolut pro' OR 'evolut pro+'/exp OR 'evolut pro+' OR 'acurate neo'/exp OR 'acurate neo' OR 'acurate neo2'/exp OR 'acurate neo2' OR 'portico'/exp OR 'portico' OR 'allegra'/exp OR 'allegra' OR 'self-expanding valve' OR 'self-expanding supraannular valve') AND ('pre dilatation' OR 'pre-dilatation' OR 'pre dilation' OR 'pre-dilation' OR 'post dilatation' OR 'post-dilatation' OR 'post dilation' OR 'post-dilation' OR 'malexpansion' OR 'incomplete expansion' OR 'underexpansion' OR 'suboptimal valve expansion' OR 'frame deformation' OR 'distorted valve frame' OR 'non-uniform deployment' OR 'irregular radial expansion') | 234 |
| **Cochrane Library** | #1 Aortic valve stenosis OR aortic stenosis; #2 Transcatheter aortic valve replacement OR TAVR OR transcatheter aortic valve implantation OR TAVI; #3 CoreValve OR Evolut OR Evolut R OR Evolut PRO OR ACURATE neo OR ACURATE neo2 OR Portico OR Allegra OR self-expanding valve OR self-expanding supraannular valve; #4 Pre dilatation OR pre-dilatation OR pre dilation OR pre-dilation OR post dilatation OR post-dilatation OR post dilation OR post-dilation OR malexpansion OR incomplete expansion OR underexpansion OR suboptimal valve expansion OR frame deformation OR distorted valve frame OR non-uniform deployment OR irregular radial expansion; #5 #1 AND #2 AND #3 AND #4 | 18 |
| **Scopus** | ( TITLE-ABS-KEY ( "Aortic valve stenosis" ) OR TITLE-ABS-KEY ( "aortic stenosis" ) ) AND ( TITLE-ABS-KEY ( "Transcatheter aortic valve replacement" ) OR TITLE-ABS-KEY ( "TAVR" ) OR TITLE-ABS-KEY ( "transcatheter aortic valve implantation" ) OR TITLE-ABS-KEY ( "TAVI" ) ) AND ( TITLE-ABS-KEY ( "CoreValve" ) OR TITLE-ABS-KEY ( "Evolut" ) OR TITLE-ABS-KEY ( "Evolut R" ) OR TITLE-ABS-KEY ( "Evolut PRO" ) OR TITLE-ABS-KEY ( "Evolut PRO+" ) OR TITLE-ABS-KEY ( "ACURATE neo" ) OR TITLE-ABS-KEY ( "ACURATE neo2" ) OR TITLE-ABS-KEY ( "Portico" ) OR TITLE-ABS-KEY ( "Allegra" ) OR TITLE-ABS-KEY ( "self-expanding valve" ) OR TITLE-ABS-KEY ( "self-expanding supraannular valve" ) ) AND ( TITLE-ABS-KEY ( "pre dilatation" ) OR TITLE-ABS-KEY ( "pre-dilatation" ) OR TITLE-ABS-KEY ( "pre dilation" ) OR TITLE-ABS-KEY ( "pre-dilation" ) OR TITLE-ABS-KEY ( "post dilatation" ) OR TITLE-ABS-KEY ( "post-dilatation" ) OR TITLE-ABS-KEY ( "post dilation" ) OR TITLE-ABS-KEY ( "post-dilation" ) OR TITLE-ABS-KEY ( "malexpansion" ) OR TITLE-ABS-KEY ( "incomplete expansion" ) OR TITLE-ABS-KEY ( "underexpansion" ) OR TITLE-ABS-KEY ( "suboptimal valve expansion" ) OR TITLE-ABS-KEY ( "frame deformation" ) OR TITLE-ABS-KEY ( "distorted valve frame" ) OR TITLE-ABS-KEY ( "non-uniform deployment" ) OR TITLE-ABS-KEY ( "irregular radial expansion" ) ) | 113 |

Supplementary Table S3: Results of Egger's regression and Begg-Mazumdar tests for publication bias in outcomes including ≥10 studies.

| **PRE-BAV Outcomes** | **No. of studies** | **Egger's regression test (p-value)** | **Begg-Mazumdar test (p-value)** |
| --- | --- | --- | --- |
| Permanent pacemaker implantation | 11 | 0.406 | 0.484 |
| Vascular complications | 11 | 0.916 | 0.586 |
| Need for POST-DIL | 10 | 0.414 | 0.531 |
| Moderate or greater paravalvular leak (PVL2+) | 11 | 0.073 | 0.139 |

Supplementary Figure S1: Funnel plots assessing publication bias for PRE-BAV outcomes including ≥10 studies.
